# An Uncommon Presentation of Pulmonary Tularemia: A Case Report and Literature Review

**DOI:** 10.7759/cureus.30379

**Published:** 2022-10-17

**Authors:** Kelly Roth, Nikhila Chelikam, Himanshu Rathore, Subramanyam Chittivelu

**Affiliations:** 1 Pulmonary Critical Care, University of Illinois, Peoria, USA; 2 Clinical Research, Icahn School of Medicine at Mount Sinai, New York, USA; 3 Internal Medicine, OSF Saint Francis Medical Center, Peoria, USA; 4 Pulmonary and Critical Care Medicine, University of Illinois College of Medicine at Peoria/OSF Saint Francis Medical Center, Peoria, USA

**Keywords:** bioterrorism, headache, fever of unknown origin, francisella tularensis, pulmonary tularemia

## Abstract

Francisella tularensis is a re-emerging organism causing more significant outbreaks of tularemia and fear of bioterrorism. It can be challenging to recognize tularemia due to its variable presentation, especially in low-incidence areas. Physicians must be mindful of this life-threatening infectious disease and consider it a differential diagnosis in patients with fever of unknown origin. We encountered a case of pulmonary tularemia with a unique presentation of severe headache and fever.

## Introduction

Francisella tularensis is a gram-negative coccobacillus causing a life-threatening zoonotic disease, tularemia [1]. Tularemia has been classified as a Category A bioterrorism disease, along with anthrax, botulism, plague, smallpox, and viral hemorrhagic fevers [2]. As few as 10-25 F. tularensis bacteria are enough to cause human disease via cutaneous or inhalation routes [3,4]. Furthermore, given its non-specific clinical presentation, diagnosis can be challenging. These factors make tularemia a budding public health crisis. We present a patient with a delayed diagnosis of pulmonary tularemia in the setting of severe headaches and fever of unknown origin.

## Case presentation

We present a 56-year-old Hispanic man, a healthy former smoker who quit several years ago with arthritis managed by a combination of hydrocodone/acetaminophen. The patient was asymptomatic until two weeks before admission. He developed low-grade fevers and fatigue for one week, followed by high-grade fever for another week. Pertinent negative history includes no cough, no urinary symptoms, no diarrhea, nausea, or vomiting. On admission, he reported a severe headache on the left side radiating to the face, behind the ear, and the temporal area. The headache was not relieved by acetaminophen or ibuprofen. He reported associated photophobia and 15 episodes of vomiting. He had six to seven episodes of drenching night sweats for the past two weeks. He is a sewage system employer by profession. He had no sick contacts and had not traveled outside Illinois in the past 17 years. He was in a monogamous, sexually active relationship with his wife. There was no family history of cancer or an autoimmune disease.

On examination, the patient appeared dehydrated and exhibited tenderness with sinus palpation. Laboratory evaluation revealed hemoconcentration and abnormal liver function studies. Urine analysis was pertinent for mild proteinuria, ketonuria, and hematuria. CSF analysis was normal. Brain MRI was unremarkable, and head CT showed mucous retention in the left sphenoid sinus with mild mucosal thickening in the ethmoid cells. The main differentials at that time were temporal arteritis, meningitis, sinusitis, migraine, medication overuse, and caffeine withdrawal. He was given bolus fluids for dehydration, and a combination of diphenhydramine, fentanyl, and 10 mg IV dexamethasone was given for his headache.

**Figure 1:**
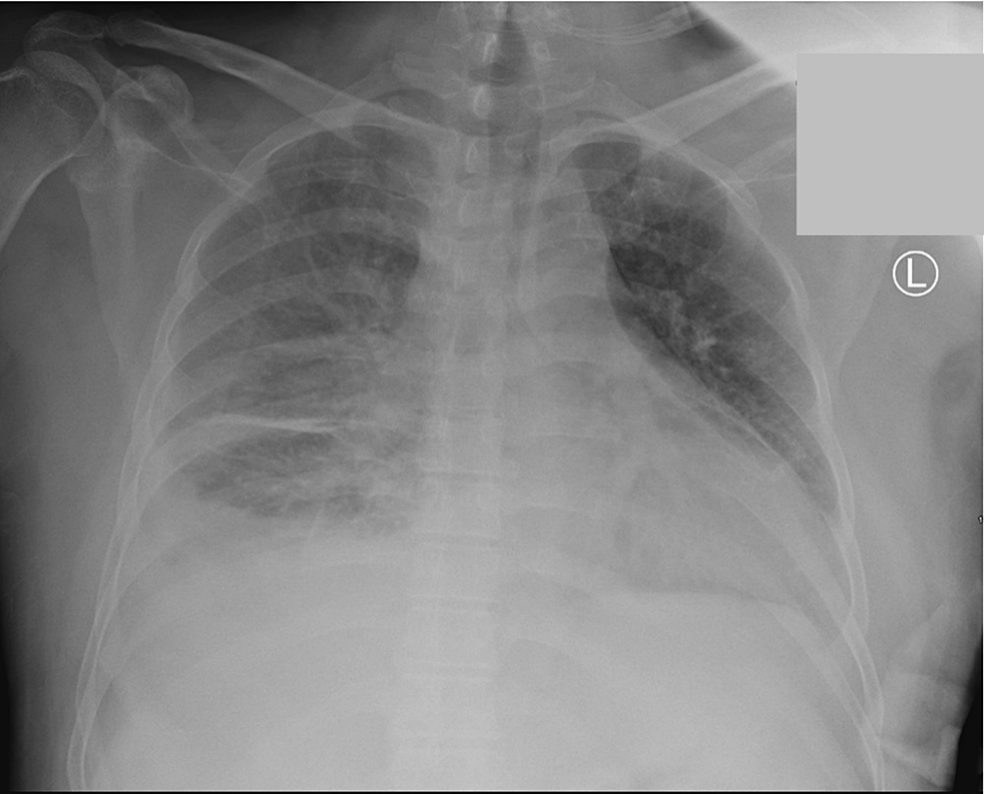
Chest X-ray showing bilateral interstitial infiltrates and right-sided pleural effusion

**Figure 2:**
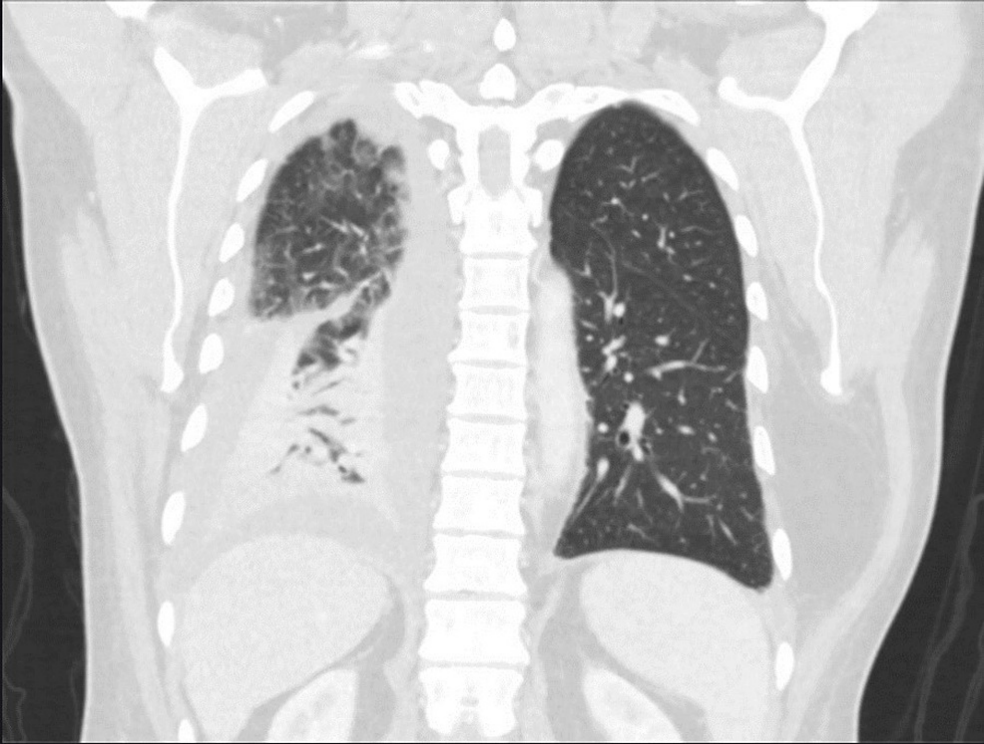
CT chest showing right lower lobe pneumonia with associated moderate-sized pleural effusion

On the second day of admission, he developed a sore throat and intermittent productive cough with thin watery sputum. The patient was desaturated with oxygen saturation (SpO2) in the 80s. He was immediately started on nasal cannula oxygen. Chest imaging was pursued due to the new onset of hypoxia. Chest X-ray showed bilateral interstitial infiltrates and right-sided pleural effusion (Figure 1). Chest CT revealed right lower lobe pneumonia with associated moderate-sized pleural effusion (Figure 2). Echocardiogram conducted showed grade II diastolic dysfunction and mild concentric LV hypertrophy with a normal ejection fraction. Diagnostic/therapeutic thoracentesis was performed, and 800 mL of pleural fluid was removed; the fluid was cloudy and further analysis revealed an exudative effusion with LDH >1900 U/L and a protein of 3 g/dL. Serum lactate dehydrogenase (LDH) was 386 U/L, and protein was 6.1 g/dL. Serum inflammatory markers were severely elevated at C-reactive protein (CRP) of 23mg/L and erythrocyte sedimentation rate (ESR) of 55mm/hr, but the other rheumatologic workup was negative. Serum inflammatory markers were severely elevated at CRP of 23 and ESR of 55, but the other rheumatologic workup was negative. A temporal artery biopsy was pursued and found to be negative. Given his persistent and worsening symptoms, a broad infectious disease panel was ordered. The infectious workup came back negative for HIV, histoplasmosis, blastomycosis, Aspergillus, toxoplasmosis, tuberculosis, gastrointestinal pathogens, Legionella, Beta-d glucan, and group A streptococcus. Doxycycline 100 mg bid was started empirically for pneumonia. Eventually, the infectious workup yielded positive results for Francisella tularensis. IgM and IgG antibodies on enzyme-linked immunoassay (ELISA) were noted to be positive; this was then confirmed by positive culture from thoracentesis fluid. Thus, the patient was diagnosed with pulmonary tularemia, complicated with parapneumonic effusion requiring chest tube drainage. It is believed that the patient acquired this infection, as he is a sewage system employer by profession.

## Discussion

Tularemia disease is caused by facultative intracellular Gram-negative coccobacillus, Francisella tularensis. The worldwide incidence is unknown today because of the under-recognition and under-reporting of tularemia. In the USA, between the years 2010 and 2019, the incidence of tularemia varied from 124 to 314 cases per 100,000 residents. It is most common in the south-central United States, the Pacific Northwest, and parts of Massachusetts. The incidence in Illinois ranged from 1-10 cases per 100,000 residents for the same period [5]. Francisella tularensis can be divided into four subspecies: ssp. tularensis, holarctica, mediasiatica and novicida. Highly virulent type A strains (ssp. tularensis) are found mainly in Northern America (the USA and Canada). In contrast, the less virulent type B strains (ssp. holarctica) are present throughout the Northern Hemisphere [1]. The primary sources of human infection include rabbits, hares, and rodents [1]. Additional sources, depending on the geographic regions, can include domestic cats [1], dogs [6], and squirrels [3,7]. Transmission to humans occurs via insect and tick bites, handling infected animals, ingesting contaminated water or food, and inhaling contaminated aerosols or dust. To date, a human-to-human spread has not been documented [1]. Individuals with occupations such as veterinarians, laboratory personnel, butchers, hunters, and farmers are at high risk of acquiring tularemia [1,8]. The patient did not belong to any above occupations and worked in a sewage system department with protective equipment. The transmission mode determines the presentation of tularemia in humans and is usually associated with an acute, febrile illness (Table 1) [8].

**Table 1:** Tularemia’s various forms of manifestations in humans

Manifestation type	Presentation
Ulceroglandular	Cutaneous ulcerations at the site of an insect or tick bite and marked lymphadenopathy
Glandular	No ulcers but marked lymphadenopathy
Oculoglandular	Preauricular lymphadenopathy, and conjunctivitis
Oropharyngeal	Pharyngitis, stomatitis, and cervical lymphadenopathy
Intestinal	Vomiting, abdominal discomfort, and diarrhea
Pneumonic	Pneumonia or pleuritis
Typhoidal	Febrile illness without any early signs or symptoms

The quick dissemination capacity of Francisella tularensis accounts for its potential use as a bioterrorism agent. The incubation period is typically two to six days but can vary anywhere from 1-21 days [3,4]. Hematogenous dissemination can lead to pneumonia in 10%-15% of ulceroglandular tularemia cases and 30%-80% of typhoid cases. Primary tularemia pneumonia was assumed to be rare until the early 1980s [9]; however, a few cases of pulmonary tularemia have been described [4,6,7,10]. Pulmonary tularemia is known to present with fever, general malaise, and respiratory symptoms [4]. A mandatory neurologic and dermatologic examination for rash and a lumbar puncture could also be considered in patients with pulmonary tularemia [7]. The patient presented with a fever of unknown origin and headache with no signs of respiratory infection initially, which misled the treatment team to several other diagnoses before reaching the final diagnosis of pneumonic tularemia. Despite the absence of photophobia or neck stiffness, bacterial sepsis and meningitis were considered and ruled out immediately, given the non-specific symptoms such as high fever and headaches.

Characteristic pulmonary tularemia on chest X-ray shows bilateral opacities, hilar lymphadenopathy, and pleural effusion. A CT thorax reveals lymphadenopathy and dense lobar consolidation, usually in the periphery [4,10]. Tularemia can be distinguished from inhalational anthrax by prominent mediastinal widening on imaging and the absence of bronchospasm. Similarly, plague can be distinguished by its rapid progression to severe pneumonia, profuse watery, or purulent sputum production, respiratory failure, sepsis, and septic shock [11]. Pulmonary tularemia could also be confused with cancer-like lesions due to atypical presentation [4,10] or community-acquired pneumonia presenting with thoracic lymphadenopathy, particularly in immunocompromised patients [3]. A few cases of myocarditis associated with Francisella tularensis were also reported in endemic regions [1]. Thus, physicians should consider pulmonary tularemia as a differential diagnosis for all these conditions. Diagnostic tests for tularemia include serology, PCR of aspirate from lymph nodes, or bacterial culture. Serological testing is the most preferred method with extensive availability and high sensitivity. An initial titer of > 1:160 or a four-fold rise between initial and convalescent serology is required [8]. However, serology (IgM and IgG) turns positive only two to four weeks after infection, so a negative test needs to be repeated in case of continued clinical suspicion [4,7,12]. Due to the pathogen's virulence and the risk of laboratory-acquired infections, direct cultures are restricted to specifically equipped laboratories and require special nutrient media and prolonged incubation [1,13].

While some patients remain asymptomatic, most progress to have an acute septic reaction followed by death [8]. Pneumonic tularemia caused by inhalation exposure to Francisella tularensis carries a mortality of 30% to 60% even after proper treatment [13]. The pulmonary complications of tularemia include lung abscess, pneumonia, and acute respiratory distress syndrome (ARDS). The extra-pulmonary complications include rhabdomyolysis, renal failure, meningitis, and peritonitis [8]. Infections with subspecies Francisella tularensis subsp. tularensis are common in North America [1,7]. Aminoglycosides are used for moderate to severe disease, and ciprofloxacin or doxycycline for mild infection. Pulmonary tularemia usually does not require surgery. Surgical intervention is recommended in cases with purulent lymphadenitis [1]. Given the chest X-ray results pointing toward atypical infection, our patient was initiated on oral doxycycline empirically. Once tularemia was diagnosed, an intravenous gentamicin regimen was added throughout the four-week hospital stay. At the time of discharge, he was prescribed oral doxycycline and oral levofloxacin for four weeks with follow-up chest CT imaging. The patient was noted to have the symptoms at the follow-up visit, and his new chest CT imaging showed persistent opacifications. He was asked to continue the antibiotics and follow up by the infectious disease department.

## Conclusions

The significant difficulties in diagnosing tularemia are its complex presentation and the time taken for culture. Since tularemia is found in more animal hosts than previously known, physicians should be aware of this disease. A high clinical suspicion is always necessary despite a lack of a history of tick bites or other definitive exposure, even in non-endemic areas. Interdisciplinary coordination is required for the timely diagnosis and treatment of this disease.

## References

[1] Frischknecht M, Meier A, Mani B, Joerg L, Kim OC, Boggian K, Strahm C (2019). Tularemia: an experience of 13 cases including a rare myocarditis in a referral center in Eastern Switzerland (Central Europe) and a review of the literature. Infection.

[2] Beale SL, Zolnikov TR, Firebaugh CM (2021). A scoping review on category A agents as bioweapons. Prehosp Disaster Med.

[3] James J, Kaul DR, Goldberger ZD, Saint S, Skerrett SJ (2015). Clinical problem-solving. Back to nature. N Engl J Med.

[4] Kravdal A, Stubhaug ØO, Piekuviene R, Sandnes A (2021). Pulmonal tularemi [Article in Norwegian]. Tidsskr Nor Laegeforen.

[5] (2022). Tularemia data and surveillance. CDC. https://www.cdc.gov/tularemia/statistics/.

[6] Matyas BT, Nieder HS, Telford SR 3rd (2007). Pneumonic tularemia on Martha's Vineyard: clinical, epidemiologic, and ecological characteristics. Ann N Y Acad Sci.

[7] Foster CL, Badlam J, De Groote MA, Chan ED (2016). A 65-year-old groundskeeper with high fever, pulmonary nodules, and thoracic lymphadenopathy. Chest.

[8] Snowden J, Simonsen KA (2022). Tularemia. https://www.ncbi.nlm.nih.gov/books/NBK430905/#_NBK430905_pubdet_.

[9] (2022). Tularemic pneumonia -- Tennessee. http://www.cdc.gov.https://www.cdc.gov/mmwr/preview/mmwrhtml/00000085.htm.

[10] Fachinger P, Tini GM, Grobholz R, Gambazzi F, Fankhauser H, Irani S (2015). Pulmonary tularaemia: all that looks like cancer is not necessarily cancer - case report of four consecutive cases. BMC Pulm Med.

[11] Dennis DT, Inglesby TV, Henderson DA (2001). Tularemia as a biological weapon. Medical and public health management. JAMA.

[12] Antonitsch L, Weidinger G, Stanek G, Markowicz M (2020). Francisella tularensis as the cause of protracted fever. BMC Infect Dis.

[13] McLendon MK, Apicella MA, Allen LA (2006). Francisella tularensis: taxonomy, genetics, and Immunopathogenesis of a potential agent of biowarfare. Annu Rev Microbiol.

